# Increased and prolonged human norovirus infection in RAG2/IL2RG deficient gnotobiotic pigs with severe combined immunodeficiency

**DOI:** 10.1038/srep25222

**Published:** 2016-04-27

**Authors:** Shaohua Lei, Junghyun Ryu, Ke Wen, Erica Twitchell, Tammy Bui, Ashwin Ramesh, Mariah Weiss, Guohua Li, Helen Samuel, Sherrie Clark-Deener, Xi Jiang, Kiho Lee, Lijuan Yuan

**Affiliations:** 1 Department of Biomedical Sciences and Pathobiology, Virginia-Maryland College of Veterinary Medicine, Virginia Tech, Blacksburg, VA 24061, USA; 2 Department of Animal and Poultry Sciences, College of Agriculture and Life Sciences, Virginia Tech, Blacksburg, VA 24061, USA; 3 Department of Large Animal Clinical Sciences, Virginia-Maryland College of Veterinary Medicine, Virginia Tech, Blacksburg, VA 24061, USA; 4 Division of Infectious Diseases, Cincinnati Children’s Hospital Medical Center, Cincinnati, OH 45229, USA

## Abstract

Application of genetically engineered (GE) large animals carrying multi-allelic modifications has been hampered by low efficiency in production and extended gestation period compared to rodents. Here, we rapidly generated *RAG2*/*IL2RG* double knockout pigs using direct injection of CRISPR/Cas9 system into developing embryos. RAG2/IL2RG deficient pigs were immunodeficient, characterized by depletion of lymphocytes and either absence of or structurally abnormal immune organs. Pigs were maintained in gnotobiotic facility and evaluated for human norovirus (HuNoV) infection. HuNoV shedding lasted for 16 days in wild type pigs, compared to 27 days (until the end of trials) in RAG2/IL2RG deficient pigs. Additionally, higher HuNoV titers were detected in intestinal tissues and contents and in blood, indicating increased and prolonged HuNoV infection in RAG2/IL2RG deficient pigs and the importance of lymphocytes in HuNoV clearance. These results suggest that GE immunodeficient gnotobiotic pigs serve as a novel model for biomedical research and will facilitate HuNoV studies.

Pigs are an excellent animal model in biomedicine because of their similarity in physiology and immunology to humans^1^. For instance, disruption of IL2RG in pigs recapitulates the phenotype of X-linked severe combined immunodeficiency (SCID) patients much closer than *Il2rg* knockout rodent models^2^. RAG2/IL2RG deficient pigs would lack B cells, T cells, and natural killer cells (B/T/NK cells). By removing all major lymphocytes, they are an ideal animal model to represent SCID patients and to study virus infection and pathogenesis in immunocompromised hosts. However, generation of these SCID pigs is technically challenging because multiple modifications of the genome by genetic engineering are required. In addition, because the gestation period of pigs is 114 days and SCID pigs fail to thrive under standard housing conditions due to their immunodeficiency^2^,^3^, producing these animals through breeding would take years and be costly. Multi-allelic modifications can be made in somatic cell then GE pigs are produced through somatic cell nuclear transfer (SCNT)^4^. However, complications can occur as founder animals born through SCNT, i.e. cloning, often have developmental defects and subsequently are not ideal as an experimental model.

Recent advances in genome editing technology, especially the CRISPR/Cas9 system, allow us to generate GE pigs at higher efficiency^5^. The CRISPR/Cas9 system, which originated from the prokaryotic adaptive immune system, is effective in creating mutations on specific loci on chromosomes and the efficiency is high enough to generate mutations during embryogenesis^6^. The system is also shown to be able to generate targeted modifications *in vivo*^7^,^8^. The system has been used to generate GE pigs for various purposes such as those resistant to porcine reproductive and respiratory syndrome virus^9^ and for xenotransplantation^4^. By engineering a complementary single-stranded RNA (crRNA) with trans-activating crRNA (tracrRNA), Cas9 can be attracted to a specific position on the chromosome resulting in a double strand break (DSB) at a desired site. The DSB will then be repaired through either non-homologous end joining (NHEJ) or homologous recombination (HR) pathway. During NHEJ repair, base pair mismatches can occur, which could generate frame shifts and result in non-functional proteins. Direct injection of CRISPR/Cas9 system into developing pig embryos can disrupt multiple genes *in vitro* and the efficiency of targeting events can be at or near 100%, suggesting that the system is an ideal approach to generate GE pigs carrying multiple genome editing events as it would not require any breeding period or SCNT process to generate animals.

Human noroviruses (HuNoVs), members of the *Norovirus* genus in the *Caliciviridae* family, are the major cause of nonbacterial epidemic acute gastroenteritis worldwide^10^, especially since the introduction of rotavirus vaccines^11^,^12^. HuNoV claims over 200,000 lives in children under 5 years old, mostly in developing countries annually^13^. In the United States, HuNoV accounts for approximately 800 deaths, 21 million illness, and $284 million in healthcare costs each year^14^,^15^. HuNoV gastroenteritis is generally self-limiting, but the disease can be severe and prolonged in specific risk groups, i.e., infants, young children, elderly, and immunocompromised individuals^16^. Although vaccine candidates are under development^17^,^18^, currently no licensed vaccines or therapeutics against HuNoV gastroenteritis are available.

The lack of a robust cell culture system and a suitable animal model has been an impediment for understanding HuNoV biology and testing antiviral strategies; limited knowledge comes primarily from studies of infected human volunteers^16^, chimpanzees^19^, gnotobiotic (Gn) calves^20^, and Gn pigs^21^,^22^,^23^. Novel animal models are urgently needed to further elucidate the molecular mechanisms of HuNoV infection, replication, and host protective immunity. Both pigs and humans are omnivorous, the general anatomy and physiology of the gastrointestinal tracts of the neonatal pig and the human are very similar^24^. After rotavirus or HuNoV infection, neonatal Gn pigs develop similar pathological changes and immune responses in the small intestine as those in humans^21^,^25^,^26^. In addition, the Gn pig model is better suited than other animal models for studies of HuNoV-induced diseases in terms of oral route of infection, clinical presence of diarrhea, and viral shedding^27^. HuNoV infection can become persistent with prolonged virus shedding in immunocompromised patients, who may suffer from increasingly debilitating and life threatening gastroenteritis^28^,^29^. Therefore, SCID Gn pigs present great promise for the study of HuNoV biology and the development of therapeutic strategies for this patient cohort.

Here, we disrupted *RAG2*/*IL2RG* via direct injection of CRISPR/Cas9 system into developing embryos, and mutations on both genes were observed at 100% efficiency *in vitro* and *in vivo*. In this approach, we rapidly generated RAG2/IL2RG deficient pigs presenting SCID phenotype, including substantial depletion of B/T/NK cells in ileum and blood, either absence of or structurally abnormal thymuses, mesenteric lymph nodes (MLN), and ileal Peyer’s patches (IPP), as well as impaired production of immunoglobulins. To evaluate the application of RAG2/IL2RG deficient pigs in biomedical research, we inoculated them with HuNoV in comparison with wild type (WT) pigs under Gn condition. HuNoV shedding in WT pigs only lasted for 16 days, whereas the shedding in RAG2/IL2RG deficient pigs was as long as 27 days, and the prolonged shedding was asymptomatic and sporadic. Higher HuNoV genomic titers were detected in intestinal tissues and contents and in blood compared to WT pigs, indicating increased and prolonged HuNoV infection in RAG2/IL2RG deficient Gn pigs.

## Results

### CRISPR/Cas9 disrupt *RAG2* and *IL2RG* in early pig zygotes

**Figure 1:**
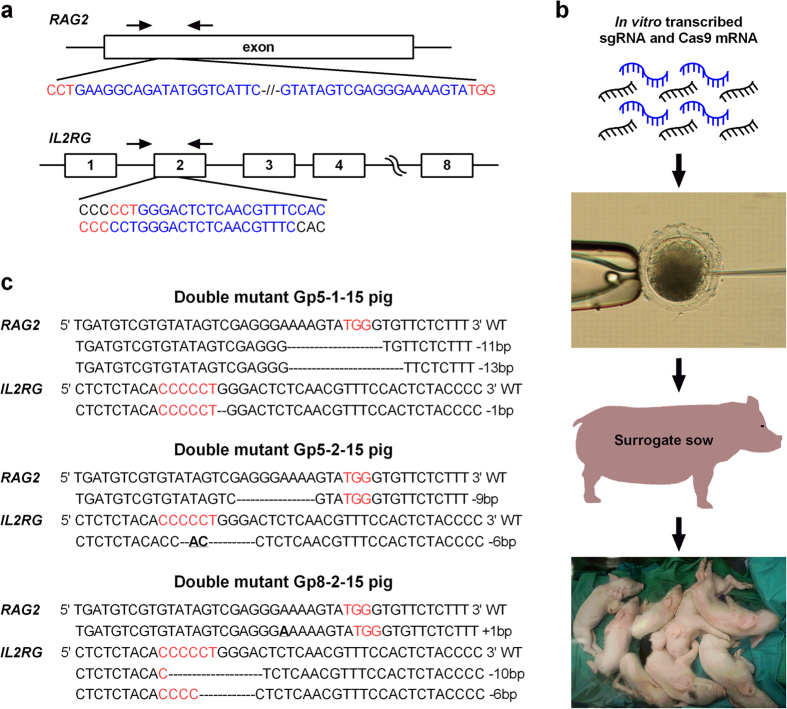
Use of CRISPR/Cas9 system to generate RAG2/IL2RG deficient pigs. (**a**) Design of CRISPRs to target *RAG2* and *IL2RG*. Sequences in blue indicate designed cRNA, letters in red reflect PAM sequences (NGG for coding and CCN for reverse strand), and arrows indicate the location of primers used to genotype embryos and piglets. (**b**) Schematic strategy. RNA form of sgRNA and Cas9 were injected into presumable zygotes at an optimized concentration. Then the injected embryos were transferred into surrogate sows. Piglets were derived by hysterectomy and maintained in Gn isolators. (**c**) Representative genotypes of RAG2/IL2RG deficient pigs. Bold letters indicate insertion or change of nucleotides, and ‘–’ indicates deletion of nucleotide. PAM sequences are highlighted in red.

CRISPR sequences were designed to specifically target Exon 1 of *RAG2* and Exon 2 of *IL2RG* (Fig. 1a). To minimize potential cytotoxicity, the CRISPR/Cas9 system was optimized to minimum concentrations of 2.5 ng μl^−1^ sgRNA and 5 ng μl^−1^ Cas9 mRNA, which introduced 100% mutation of *RAG2* on both alleles and did not impair embryo development *in vitro* (Supplementary Table 1). To verify efficacy of the CRISPR/Cas9 system in targeting *RAG2* and *IL2RG* simultaneously, embryos injected with the CRISPR/Cas9 system were genotyped on day 6 post microinjection. Mutations on both *RAG2* and *IL2RG* were observed in all 6 genotyped blastocysts, in which 5 had biallelic mutations and 1 had mosaic genotype of *RAG2*; 2 had homozygous mutations and 4 had biallelic mutations of *IL2RG* (Supplementary Table 2). Nevertheless, no wild type sequence was found from the genotyping (Supplementary Fig. 1), indicating 100% disruption of both genes and the piglets developed from the embryos would show SCID phenotype.

### Generation of RAG2/IL2RG deficient pigs

RAG2/IL2RG deficient pigs were generated by injecting CRISPR/Cas9 RNA into early *in vitro* fertilization (IVF)-derived zygotes and performing embryo transfer. Embryos were transferred into five surrogate sows, piglets were subsequently derived by hysterectomy and maintained in Gn isolators under germ-free condition (Fig. 1b). Three surrogates maintained pregnancy, from which seventeen live piglets and two stillborn were produced (Supplementary Table 3). Genotyping results obtained from newborn pig tail samples demonstrated that no pigs carried wild type sequence of either *RAG2* or *IL2RG* gene (Fig. 1c and Supplementary Table 4), consistent with our *in vitro* analysis. We were not able to amplify a fragment of *IL2RG* flanking the CRISPR/Cas9 projected cutting site for one piglet (Gp7-7-15); no fragment was detected when we expanded the amplification up to 1 kb, suggesting a larger deletion of *IL2RG* caused by the CRISPR/Cas9 system (Supplementary Fig. 2).

### SCID phenotype in RAG2/IL2RG deficient pigs

**Figure 2:**
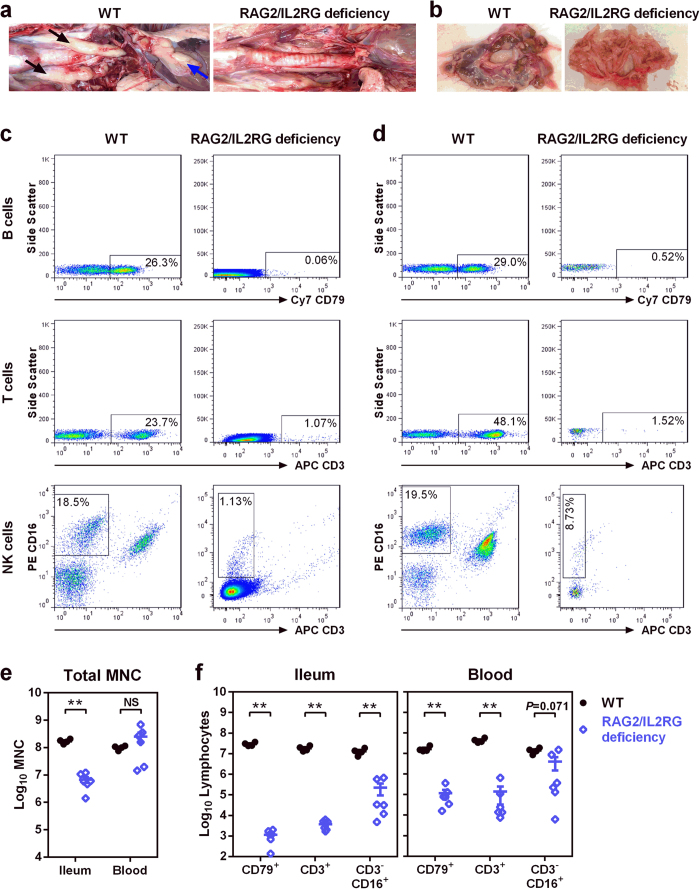
SCID phenotype of RAG2/IL2RG deficient pigs at 34 days of age. (**a**) Representative images showing thoracic thymus (black arrows) and cervical thymus (blue arrow) in WT pigs, while thoracic and cervical thymus were lacking in some RAG2/IL2RG pigs. (**b**) Representative images of the mesentery showing the lack of MLN in some RAG2/IL2RG pigs. Representative flow cytometry of MNC from ileum (**c**) and blood (**d**) showing a significant reduction of B cells (CD79^+^), T cells (CD3^+^), and NK cells (CD3^−^CD16^+^), as indicated by dot plots gated within lymphocytes. Cy7, cyanine 7; APC, allophycocyanin; PE, phycoerythrin. (**e**) Total number of MNC from 40 cm ileum and 70 ml blood were isolated and quantified for WT (*n* = 4) and RAG2/IL2RG deficient pigs (*n* = 6). (**f**) Lymphocytes in ileum and blood were quantified based on the total number of MNC and the proportion of cells within MNC (Supplementary Fig. 5). Data are presented as means ± s.e.m. with individual animal data points (**e**,**f**). Statistical significance was determined by Mann-Whitney test. NS, not significant, **P < 0.01.

Postmortem analysis of the RAG2/IL2RG deficient pigs showed clear gross or histological evidence of SCID phenotype. As summarized in Supplementary Table 5, eight pigs lacked thoracic thymus, and three lacked lymphocytes within observed thoracic thymuses; seven lacked cervical thymus, and six lacked lymphocytes within cervical thymuses; ten lacked MLN; and eleven lacked IPP (also see Fig. 2a,b and Supplementary Fig. 3). Although thymus, MLN, and/or IPP were observed in some RAG2/IL2RG deficient pigs, their morphology was abnormal compared to those of WT pigs, including the lack of corticomedullary distinction in thymuses, the absence of follicles in MLN, as well as poorly developed and unstructured small IPP (Supplementary Fig. 4).

To characterize the immune system of RAG2/IL2RG deficient pigs, especially the intestinal immune cells, ileum and circulating blood from each pig were collected for the isolation of mononuclear cells (MNC). Compared to WT pigs, flow cytometry analysis showed that their B/T/NK cells were substantially depleted in ileum and blood (Fig. 2c,d). The total number of MNC was about 10-fold lower in the ileum (Fig. 2e). Together with the proportion of B/T/NK cells within MNC (Supplementary Fig. 5), the total number of B and T cells were significantly lower in ileum and blood (Fig. 2f). The significant difference of NK cells was observed in ileum, but not in blood (Fig. 2f), because two out of six measured pigs possessed hypomorphic IL2RG mutations, resulting in partial disruption of IL2RG and incomplete depletion of NK cells (Supplementary Tables 4 and 5). To confirm the depletion of B cells in RAG2/IL2RG deficient pigs, total immunoglobulin titers in serum were measured. IgA was undetectable, while IgG and IgM were significantly lower than those of WT pigs (Supplementary Fig. 6a). In addition, B cells were not observed in mesentery tissues by immunohistostaining targeting cellular marker CD79 (Supplementary Fig. 6b). In all, the absence of or structurally abnormal immune organs and depletion of lymphocytes indicated SCID phenotype in RAG2/IL2RG deficient pigs, making these pigs a novel large animal model for the studies of viral pathogenesis and immunity in immunocompromised hosts.

### Increased and prolonged HuNoV shedding in SCID Gn pigs

One RAG2/IL2RG deficient Gn pig was euthanized at 3 days of age due to general weakness rather than illness caused by pathogens; and one was used as a mock infection control. As HBGA type A^+^ or H^+^ hosts are more susceptible to HuNoV infection^30^,^31^,^32^, all pigs (including a control group of WT Gn pigs) in this study were confirmed A^+^ or H^+^ (Supplementary Fig. 7 and Supplementary Table 5). The pigs were inoculated at 6 or 7 days of age with a HuNoV GII.4/2006b variant, which has been previously characterized in WT Gn pigs^22^, and then euthanized on post inoculation day (PID) 3, PID10, or PID28. The effects of RAG2/IL2RG deficiency on HuNoV-induced disease and infectivity were evaluated daily by comparing diarrhea parameters and fecal virus shedding to WT pigs.

**Figure 3:**
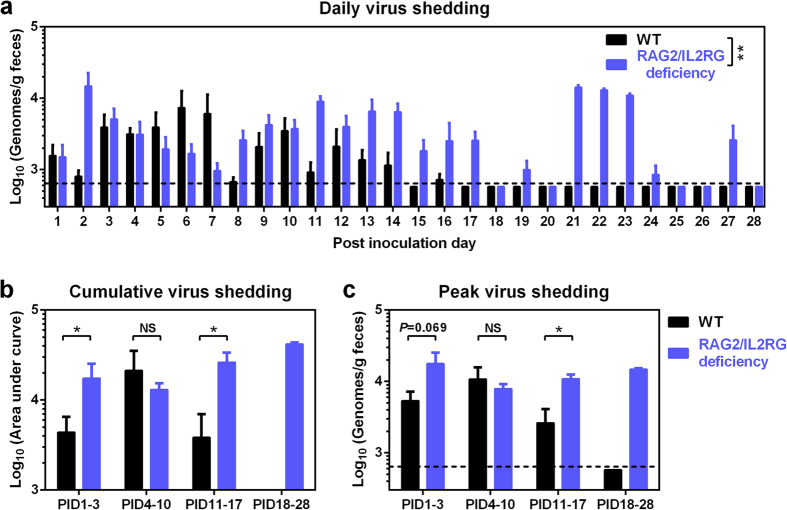
Increased and prolonged fecal HuNoV shedding in RAG2/IL2RG deficient pigs. (**a**) Daily virus shedding was monitored from PID1 to PID28 by rectal swab sampling of feces and quantitative reverse transcription polymerase chain reaction (qRT-PCR) to quantify the HuNoV genomes. (**b**) Individual pigs’ cumulative virus shedding was presented as area under curve from (**a**). (**c**) Peak virus shedding titers from PID1 to PID3, PID4 to PID10, PID11 to PID17, and PID18 to PID28 in individual pigs. Sample sizes are shown in Table 1. Dashed line indicates limit of detection. Data are presented as mean ± s.e.m. Statistical significance was determined by two-way analysis of variance (ANOVA) (**a**) or Mann-Whitney test (**b**,**c**). NS, not significant, *P < 0.05, **P < 0.01.

As a self-limiting enteric virus, HuNoV shedding only lasted for 16 days in WT pigs, peaking on PID6 and PID10 (Fig. 3a). In contrast, HuNoV shedding could be detected for as long as 27 days in RAG2/IL2RG deficient pigs, peaking on PID2, PID11, and PID21-23 (Fig. 3a). RAG2/IL2RG deficient pigs had significantly longer duration of virus shedding than WT pigs on PID11–17 and PID18–28 (5.5 versus 1.2 days and 4.5 versus 0 days, respectively), and all RAG2/IL2RG deficient pigs had shedding on PID18–28 (Table 1), indicating prolonged HuNoV infection in SCID pigs. However, no significant difference in the incidence of diarrhea was observed for the two groups at any time points (Table 1), indicating that the prolonged HuNoV infection and fecal shedding in SCID pigs were not associated with increased duration of diarrhea, i.e., it was asymptomatic. Compared to WT pigs, the cumulative virus shedding in RAG2/IL2RG deficient pigs was significantly higher on PID1–3 and PID11–17 (Fig. 3b). There was also a trend for higher peak shedding titer on PID1–3 and significantly higher titer on PID11–17 (Fig. 3c), altogether indicating increased HuNoV replication in SCID pigs.

### HuNoV distribution in Gn pigs

**Figure 4:**
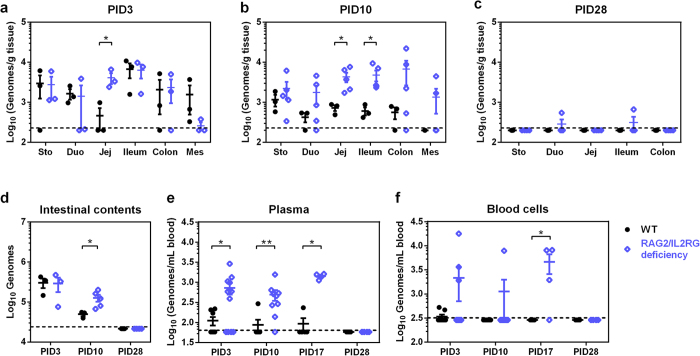
HuNoV distribution in Gn pigs. HuNoV genomes in stomach (Sto), duodenum (Duo), jejunum (Jej), ileum, and mesentery (Mes) from pigs euthanized on PID3 (**a**), PID10 (**b**), and PID28 (**c**) were measured by qRT-PCR. (**d**) Total HuNoV genomes in intestinal contents were measured by qRT-PCR. HuNoV genomes in plasma (**e**) and whole blood cells (**f**) were measured by qRT-PCR. (**a**–**d**) WT groups, PID3 *n* = 3, PID10 *n* = 3, PID *n* = 5; RAG2/IL2RG deficiency groups, PID3 *n* = 3, PID10 *n* = 5, PID *n* = 4. (**e**,**f**) Sample sizes are shown in Table 1. Dashed line indicates limit of detection. Data are presented as mean ± s.e.m. with individual animal data points. Statistical significance was determined by Student’s *t*-test (**a**) or Mann-Whitney test (**b**–**f**). *P < 0.05, **P < 0.01.

HuNoV (GII.4) antigen was observed previously in enterocytes of WT Gn pigs^21^,^22^,^23^. In this study, both WT and RAG2/IL2RG deficient pigs had confirmed HuNoV infection in enterocytes of duodenum and jejunum (Supplementary Fig. 8). Analysis of gut tissues indicated the existence of HuNoV genomes in all sections of the gastrointestinal tract of both WT and RAG2/IL2RG deficient pigs. Overall, virus titers in gut tissues of WT pigs peaked on PID3 and declined overtime as expected, but virus titers remained consistent in RAG2/IL2RG deficient pigs from PID3 to PID10 (Fig. 4a,b). Specifically, in RAG2/IL2RG deficient pigs, virus titers were significantly higher in jejunum on PID3 (Fig. 4a), and significantly higher in jejunum and ileum on PID10 than in WT pigs (Fig. 4b). HuNoV genomes were detectable in 2 of 4 RAG2/IL2RG deficient pigs on PID28, but not in WT pigs (Fig. 4c). HuNoV genomes were present in many extraintestinal tissues, and the titers peaked on PID10 for both pig groups (Supplementary Fig. 9). Similar to the findings from clinical human samples^33^, HuNoV genomes were consistently undetectable in cerebrospinal fluid (CSF) in this study, suggesting that HuNoV is blocked by the blood-brain barrier of WT and SCID hosts. Unlike WT pigs, HuNoV genomes were detected in liver of RAG2/IL2RG deficient pigs on PID10, presumably due to loss of protection following the depletion of lymphocytes.

The entire contents of small and large intestine were collected during necropsies and virus titers were determined. HuNoV titer was significantly higher in RAG2/IL2RG deficient pigs on PID10 compared to WT pigs (Fig. 4d). Transient HuNoV viremia was observed previously in Gn pigs and calves^20^,^21^. In this study, virus titers in plasma were significantly higher in RAG2/IL2RG deficient pigs on PID3, PID10, and PID17, whereas HuNoV genomes were not detectable on PID28 from either group (Fig. 4e). Similarly, HuNoV genomes were detectable in whole blood cells, and the titer was significantly higher in RAG2/IL2RG deficient pigs on PID17 (Fig. 4f). Taken together, increased and prolonged HuNoV titers in intestinal tissues and contents and in blood demonstrated that RAG2/IL2RG deficiency promoted HuNoV infection of Gn pigs. Thus, B/T/NK lymphocytes are important in the control and clearance of HuNoV infection.

## Discussion

Conventionally, GE pigs with specific genetic modifications are generated by engineering somatic cells then performing SCNT^34^,^35^. This approach has been successfully applied to generate GE pigs, including pigs carrying multi-allelic modification^4^,^34^. However, GE founder pigs produced by this approach often present unexpected phenotypes due to the cloning process^36^. Therefore, progeny of the founder animals is generally used for experiments. Considering the gestation period and days to reach puberty in pigs, this can easily take 1–2 years. The extended time required to generate GE animals and higher housing costs have been the biggest challenges in using GE pig models in biomedicine. Our data showed that the CRISPR/Cas9 system could modify *RAG2* and *IL2RG* simultaneously with 100% efficiency *in vitro* and *in vivo* when introduced into early embryos, demonstrating that the technology is effective and bypasses the need of cloning in generating GE pigs carrying multi-allelic modifications, the RAG2/IL2RG deficient pigs. Because all animals generated through this approach carried the modification, no breeding was required to propagate GE founders to obtain enough animals, thereby reducing the time required to generate GE large animals such as pigs; the entire process took less than six months. There are reports of successfully utilizing CRISPR/Cas9 system to introduce specific mutation during embryogenesis in pigs^37^,^38^,^39^, but to our best knowledge, this is the first report of whole animal multiplexing in non-rodent species at the efficiency of 100% by using the system during embryogenesis. Furthermore, although different SCID pigs have been reported previously^2^,^3^,^40^, this is the first report of large animal model that lacks B/T/NK cells.

Mosaic genotype can be a major concern in using CRISPR/Cas9 technology^6^,^41^; however, we had only one pig for *RAG2* and two pigs for *IL2RG* out of the seventeen with mosaic genotype, and no wild type sequence was found (Supplementary Table 5), indicating that most of the modifications through CRISPR/Cas9 system were made at the one-cell stage. Considering the average number of piglets in a sow (seventeen piglets from three sows), the frequency of mosaic genotype should not be a concern in generating GE pigs via this methodology. The rapid generation of multi-allelic mutant pigs is a clear advantage of this approach, though a potential side effect involves genetic variance among piglets derived from each batch of CRISPR/Cas9 injections as oocytes are supplied from a local slaughterhouse. This genetic variance can potentially introduce a higher variation to the data collected using the animals as a model.

We could not identify modification of *IL2RG* at the nucleotide level in one SCID piglet we produced in this study. It appeared to have a large deletion on the *IL2RG*. The CRISPR/Cas9 system is known to generally induce smaller insertions or deletions, but it is also known to introduce unexpected large deletions^39^. These larger deletions are difficult to identify by the PCR approach, which may interfere with genotyping animals at the nucleotide level. In a previous study, any change in *RAG2* was sufficient to inactivate its function in pigs^3^. This was consistent in our study; premature stop codon, generated by the CRISPR/Cas9 system was not required to inactivate its function. On the other hand, hypomorphic mutation of *IL2RG* did not fully disrupt its function as NK cells were detected in blood from those pigs in this study. All those pigs carried changes in nucleotide in triplets, indicating that a premature stop-codon was not generated by frameshift mutation. Targeted mutations through NHEJ are random, so loss or addition of nucleotides in triplets often does not fully inactivate the protein. This can be a potential side effect of generating GE animals through the direct injection of CRISPR/Cas9 systems into zygotes. Potentially, using HR directed repair could ensure the appearance of premature stop codon, though the approach is not very effective during embryogenesis in pigs. Nonetheless, ileal NK cells in those pigs with hypomorphic mutation of IL2RG were still significantly depleted, thus they were usable for our study of HuNoV infection.

HuNoV gastroenteritis is characteristically acute and self-limiting with a duration of 24 to 72 hours, but in immunocompromised patients, the infection and disease can become persistent for weeks to years, and the chronic viral enteritis can become debilitating and life-threatening^28^,^29^. There is currently no virus-specific therapy available for HuNoV infection, although such therapies are in greater demand in immunocompromised populations^42^. Balb/c RAG/IL2RG deficient mice support HuNoV infection, but the duration was only three days, and fecal virus shedding and gastrointestinal disease were not observed. Thus, this mouse model does not reflect HuNoV biology as in immunocompromised humans^43^. Among the related murine noroviruses (MNVs), MNV-1 can establish persistent infection in RAG1 deficient mice, which lack B and T cells^44^. Here, we demonstrated that RAG2/IL2RG deficient Gn pigs supported increased and prolonged HuNoV infection. Higher virus shedding was observed for the first 3 days, indicating SCID hosts were more susceptible to HuNoV infection at the initial stage. Furthermore, the prolonged HuNoV shedding in SCID pigs (after PID17) was asymptomatic and sporadic, which is similar to asymptomatic low virus shedding in immunosuppressed patients^45^. Fecal HuNoV shedding in WT Gn pigs had two clusters peaking on PID6 and PID10, but virus shedding in RAG2/IL2RG deficient Gn pigs had three clusters peaking on PID2, PID11, and PID21-23. The differences in kinetics might result from the higher susceptibility and rapid evolution of HuNoV in SCID hosts^29^,^46^, and the rapid evolution might also attribute to sporadic virus shedding after PID17, such as on PID27.

B cells were shown to be susceptible to HuNoV infection *in vitro* (human BJAB cell line) and in chimpanzees^19^,^47^,^48^, but enterocytes are the only cell type that has been observed with HuNoV infection in Gn pigs to date^21^,^22^,^23^. Unlike the reduced MNV-3 titers in RAG1 deficient mice and B cell deficient mice compared to WT mice^49^, increased HuNoV titers were observed in RAG2/IL2RG deficient pigs in this study, suggesting that B cells might not be the target cell type of HuNoV in Gn pigs. HuNoV genomes were detected in stomach tissue in both groups with similar titers, presumably accumulating from duodenum-derived virions by reverse peristalsis rather than viral infection and replication in the stomach. HuNoV genomes were also detected in extraintestinal regions, but the titers peaked on PID10 for both groups and it is likely those are virions that translocated through circulating blood. The liver is a critical and unique organ populated with immune cells to eliminate pathogens in blood^50^, the lack of lymphocytes may explain how HuNoV genomes were detected in SCID pigs, but were consistently negative in WT pigs. As HuNoV genomes were only detected at low levels in duodenum, ileum, and spleen from 2 of 4 SCID pigs on PID28, it is likely that HuNoV infection would not persist, and that fecal shedding afterward, if there were any, would be asymptomatic and sporadic with low viral loads.

Pig models, especially SCID models, are valuable in studying pathogenesis of human pathogens because of similarities in immunity and physiology between pigs and humans. The importance of these large animal models is increasingly being recognized by the biomedical research community^51^,^52^. However, due to low efficiency in production and difficulty in housing the SCID pigs long-term for breeding, their application has been limited. In this study, we introduced a novel experimental platform to apply SCID pigs in pathogenesis study by using CRISPR/Cas9 and Gn systems. The CRISPR/Cas9 system allows us to generate multi-allelic mutant pigs without cloning or breeding, and the Gn system can protect the pigs from other pathogens. Through our study, we found that SCID Gn pigs infected with HuNoV had increased and prolonged infection when compared to immunocompetent WT Gn pigs. The combination of the CRISPR/Cas9 system and gnotobiology demonstrates an ideal platform to use SCID pigs in biomedical research.

## Methods

### Virus

Stool containing HuNoV GII.4/2006b variant 092895 (GenBank no. KC990829) was collected at Cincinnati Children’s Hospital Medical Center from a child with norovirus gastroenteritis in 2008. The protocol for stool sample collection was approved by the institutional review boards of the Cincinnati Children’s Hospital Medical Center (IRB number: 2008–1131), and informed consent was obtained from parents or child for future studies; the sample collection procedures were carried out in accordance with the approved guidelines. The stool was processed as inoculum and stored in our laboratory^22^. Inoculum was tested by culturing in thioglycollate medium and blood-agar plates to confirm sterility, and absence of other viruses was confirmed by a Virochip Microarray (University of California, San Francisco, Viral Diagnostics and Discovery Center).

### CRISPR/Cas9 system to target *RAG2/IL2RG*

To disrupt *RAG2* and *IL2RG*, a total of four sgRNAs (two for each gene) were designed. A web-based program was used to design the target sequences (http://www.crispr-cas.org/p/resources.html), and then the designed sgRNA sequences were blasted against entire pig genome for specificity. The selected primers were annealed and ligated into the px330 vector^53^. The RNA form of CRISPR/Cas9 system was synthesized by *in vitro* transcription as previously described^39^ (primers in Supplementary Table 6).

### *In vitro* fertilization (IVF)

Pig ovaries were collected at a local abattoir, or sow-derived oocytes were purchased (DeSoto, Inc). Medium-sized follicles from ovaries transported from a local abattoir were aspirated by 18-gage needle attached to a 10 ml sterile syringe. Cumulus oocyte complex (COC) were maturated *in vitro* in a TCM-199 based maturation media containing 0.5 IU ml^−1^ FSH, 0.5 IU ml^−1^ LH, 0.82 mM cysteine, 3.02 mM glucose, 0.91 mM sodium pyruvate, and 10 ng ml^−1^ EGF. After 42–44 hours of maturation at 38.5 °C and 5% CO_2_, cumulus cells were removed by exposing the oocytes into a media containing 0.1% hyaluronidase. Oocytes that extruded the first polar body were used for IVF. Then mature oocytes, groups of 25–30 oocytes, were placed in 50 μl droplets of IVF medium (modified Tris-buffered medium with 113.1 mM NaCl, 3 mM KCl, 7.5 mM CaCl_2_, 11 mM glucose, 20 mM Tris, 2 mM caffeine, 5 mM sodium pyruvate, and 2 mg ml^−1^ BSA) and covered with mineral oil. Extended semen was washed with PBS three times. After the wash, sperm pellet was resuspended with mTBM media. Then, 50 μl sperm (2.5 × 10^5^ sperm ml^−1^) was introduced into mTBM drops that contained oocytes. The gametes were co-incubated for 5 hours at 38.5 °C and 5% CO_2_. Presumably fertilized embryos were then placed in Porcine Zygote Media 3 (PZM-3)^54^ at 38.5 °C, 5% CO_2_, and 5% O_2_ incubator until microinjection of CRISPR/Cas9 system.

### Microinjection of CRISPR/Cas9 system

After 2 hours post-IVF, presumable zygotes were injected with CRISPR/Cas9 system to disrupt *RAG2*/*IL2RG*. Concentration of 2.5 ng μl^−1^ sgRNA and 5 ng μl^−1^ Cas9 mRNA was injected into the cytoplasm of fertilized oocytes using a FemtoJet microinjector (Eppendorf, Hamburg, Germany). Microinjection was conducted in manipulation medium (TCM199 with 0.6 mM NaHCO_3_, 2.9 mM HEPES, 30 mM NaCl, 10 ng ml^−1^ gentamicin, and 3 mg ml^−1^ BSA) on the heated stage of a Nikon inverted microscope (Nikon Corporation, Tokyo, Japan). Injected zygotes were then transferred and cultured into the PZM-3. Embryos used for embryo transfer were cultured in PZM-3 in the presence of 10 ng ml^−1^ GM-CSF^55^.

### Genotyping the mutations generated by CRISPR/Cas9 system

To amplify the target region of *RAG2* and *IL2RG*, primers were designed flanking projected double strand break (DSB) sites (Supplementary Table 6). DNA was extracted from a single embryo by incubating an individual embryo in the embryo lysis buffer (50 mM KCl, 1.5 mM MgCl_2_, 10 mM Tris-HCl pH 8.5, 0.5% Nonidet P40, 0.5% Tween-20 and 200 μg ml^−1^ proteinase K) at 65 °C for 30 min followed by 95 °C for 10 min. Genomic DNA from pigs was isolated using PureLink Genomic DNA kit (Thermo Fisher Scientific) following the manufacturer’s instructions. The target regions were amplified by using Platinum Taq DNA Polymerase (Thermo Fisher Scientific). PCR conditions were as follows, initial denature at 95 °C for 2 min, denature at 95 °C for 30 sec, annealing at 55 °C for 30 sec and extension at 72 °C for 30 sec for 34 cycles, 72 °C for 5 min and holding at 4 °C. PCR amplicons were sequenced to verify mutations generated by introduced CRISPR/Cas9 system.

### Surgical embryo transfer

IVF embryos, injected with CRISPR/Cas9 system, were transferred into a total of five surrogate sows at day 5 or 6 post-IVF. The embryos were surgically transferred deep into the oviduct of the sows. Pregnancy was determined by ultrasound at day 30 of gestation.

### Gnotobiotic pigs and HuNoV inoculation

Near-term WT and SCID pigs (Yorkshire cross breed) were derived via hysterectomy and maintained in Gn pig isolators. Sterility was monitored weekly by culturing feces from rectal swabs in thioglycollate medium and blood-agar plates as described previously^22^,^56^. Pigs were orally inoculated at 6–7 days of age with 10 ID_50_ of HuNoV (2.74 × 10^4^ viral RNA copies)^22^. Four ml of 200 mM sodium bicarbonate was given 15 min prior to inoculation to neutralize stomach acids. Clinical signs and virus shedding were monitored daily until euthanasia on PID3, PID10, or PID28 for collection of blood, intestinal contents, and tissues. All animal experimental protocols were approved by the Institutional Animal Care and Use Committee at Virginia Tech (IACUC protocol: 14-108-CVM). All experimental procedures were carried out in accordance with federal and university guidelines.

### HBGA typing

Pigs were blood-typed by PCR and/or immunofluorescence assay (Supplementary Fig. 7), HBGA type A^−^ and H^−^ pigs were excluded from this study. For PCR blood typing, genomic DNA was extracted from 20 μl whole blood using DNAzol Genomic DNA Isolation Reagent (Molecular Research Center) following the manufacturer’s instructions. A^+^ or A^−^ was determined by a 500 bp PCR product using forward primer ABO4s and reverse primer ABO5a, while primers Pig5 and Pig3 were used as internal control (Supplementary Table 6). For immunofluorescence assay^22^, pig cheek swabs were collected and swirled in PBS, in which buccal cells were spun down and washed with PBS, and resuspended in 20 μl PBS. 2 μl buccal cell suspension was air dried on slides and fixed in cold acetone. HBGA phenotypes were measured by blood group A antigen antibody (sc-69951; Santa Cruz; 1:500), blood group H antigen antibody (sc-52369; Santa Cruz; 1:500), and AlexaFluor 488-labled secondary antibody (A-10680; Thermo Fisher Scientific; 1:500). Slides were mounted in Vectashield containing 4,6-diamidino-2-phenylindole (DAPI) to stain cell nuclei (Vector Laboratories).

### Isolation of MNC

Ileum (40 cm) and blood (70 ml) were collected during necropsies for the isolation of MNC as described previously^26^. Briefly, segments of ileum were rinsed with wash medium and Hanks Balanced Salt Solution without Ca^2+^ and Mg^2+^ (HBSS/Modified; GE Healthcare), and intraepithelial lymphocytes and epithelial cells were dislodged mechanically by horizontal rotation. Segments were minced, resuspended in RPMI-1640 medium containing 10% fetal bovine serum and 400 U ml^−1^ of type 2 collagenase (Worthington Biochemical Co), and digested at 37 °C for 30 min with gentle shaking. The supernatants were then collected, and the remaining tissues were ground on an 80-mesh screen to obtain single cell suspensions. Cells pooled from supernatants and suspensions above were resuspended to a final concentration of 30% percoll (GE Healthcare) and centrifuged at 1800*g* for 20 min at 4 °C to remove mucous. Cell pellets were resuspended in 43% Percoll, underlaid with 70% Percoll, and centrifuged at 1800*g* for 30 min at 4 °C. MNC were collected from the 43% to 70% Percoll interface. Blood was collected in 30% (vol/vol) acid citrate dextrose, and peripheral blood lymphocytes (PBL) were isolated by Ficoll-Paque PREMIUM (GE Healthcare) density gradient centrifugation at 1200 *g* for 30 min. PBL were collected from the interface and washed in distilled H_2_O for 5 to 10 sec to lyse the remaining red blood cells.

### Flow cytometry

For detecting CD3^+^ and CD3^−^CD16^+^ lymphocytes, 2 × 10^6^ of MNC were stained at 4 °C for 15 min in 100 μl of staining buffer with 2 μl mouse (IgG1) anti-pig CD3ε (4510-01; Southern Biotech) and 1 μl Phycoerythrin (PE) conjugated mouse (IgG1) anti-pig CD16 (MCA1971PE; AbDSerotec), followed by 1 μl Allophycocyanin conjugated rat (IgG1) anti-mouse IgG1 (A85-1; BD Pharmingen). Cells were fixed/permeabilized with BD cytofix/cytoperm™ buffer (BD pharmingen) at 4 °C for 30 min. One ml of staining buffer (prepared according to BD Pharmingen™ BrdU Flow Kits Instruction Manual) was used to wash cells between steps and cells were centrifuged at 500*g* for 5 min at 4 °C. For detecting the frequencies of CD79^+^ lymphocytes among MNC, cells were first fixed/permeabilized with BD cytofix/cytoperm™ buffer at 4 °C for 30 min, then stained at 4 °C for 30 min in 100 μl of BD perm/wash buffer (BD pharmingen) with 0.5 μl mouse (IgG2b) anti-human CD79a (Vp-C366; Vector Laboratories), followed by stained at 4 °C for 30 min in 100 μl of BD perm/wash buffer with 0.5 μl PE-Cy7 goat anti-mouse IgG2b (1090-17, SouthernBiotech). One ml of BD perm/wash buffer was used to wash cells between steps and cells were centrifuged at 500*g* for 5 min at 4 °C, followed by washing with 1 ml of staining buffer. At least 20,000 cells were acquired on a FACSAria flow cytometer (BD Biosciences). Flow cytometry data were analyzed using FlowJo 7.2.2 software (Tree Star, Ashland, Oregon). The absolute numbers of CD3^+^, CD3^−^CD16^+^ and CD79^+^ lymphocytes per tissue were calculated based on their frequencies among MNC and the total number of MNC isolated from each tissue.

### ELISA for total immunoglobulin

Total immunoglobulin (Ig) titers in serum were measured by ELISA. 96-well plates were coated with goat anti-porcine IgM (01-14-03; KPL; 15 μg ml^−1^), IgA (A100-102A; Bethyl; 18 μg ml^−1^), or IgG (01-14-02; KPL; 3 μg ml^−1^) antibody overnight at 4 °C. Plates were washed twice with PBS containing 0.05% Tween-20 (PBST), blocked with PBST containing 2% non-fat milk at 37 °C for 2 h, and washed twice with PBST. Sera were serially diluted with PBST containing 2% non-fat milk, and incubated on coated plates at 37 °C for 1 h. After washing for four times, horseradish peroxidase-conjugated goat anti-porcine IgM (04-14-03; KPL; 1:5000), IgA (A100-102p; Bethyl; 1:5000), or IgG (A100-104p; Bethyl; 1:500) antibody was added and incubated at room temperature for 1h. Plates were washed four times with PBST and developed by ABTS substrate (KPL). The highest sample dilution that produced a mean absorbance (A_405_) greater than the cut-off value (mean A_405_ of negative controls plus three times of standard deviation) was considered as the Ig titer.

### Detection of HuNoV by qRT-PCR

HuNoV genomes were detected by a one-step TaqMan qRT-PCR, for which RNA templates from different samples were processed as follows. To assess virus shedding, pig feces were collected daily following HuNoV inoculation by rectal swab sampling, then rectal swabs were swirled in 1 ml PBS to release feces, after vortexing and centrifugation at 10000*g* for 5 min, 250 μl supernatant was prepared for total RNA isolation. Gut tissues were collected during necropsies and immediately frozen in liquid nitrogen; 40 to 60 mg of tissues were washed in PBS and homogenized in 0.2 ml TRIzol LS (Thermo Fisher Scientific) with 100 mg of 1.0 mm Zirconium Oxide beads (Next Advance) using Bullet Blender (Next Advance) for 10 min at maximum speed, and RNA was isolated by adding another 0.55 ml TRIzol LS. Intestinal contents were collected and the total volume was measured. Five ml were prepared as 10% solution using diluent #5 (Minimal Essential Medium with 1% penicillin-streptomycin and 1% HEPES), and RNA was isolated from 100 μl diluted intestinal contents. Plasma and whole blood cells were separated from blood containing ACD by centrifugation at 2000 *g* for 5 min. Plasma from 250 μl blood was used for RNA isolation, and whole blood cells from 50 μl blood was washed in PBS and used for RNA isolation. MNC from ileum were isolated as described above and 2 × 10^6^ cells were used for RNA isolation.

Samples processed above were isolated for total RNA using 750 μl TRIzol LS following the manufacturer’s instructions. The RNA was dissolved in 40 μl DEPC-treated water, and 5 μl RNA was used for the 25 μl qRT-PCR reaction with a SensiFAST Probe No-ROX One-Step Kit (Bioline) to detect HuNoV genomes. Primers COG2F, COG2R, and probe RING2 were adapted from a previous study (Supplementary Table 6)^57^. Cycling conditions were: reverse transcription at 45 °C for 10 min, polymerase activation at 95 °C for 2 min, 40 cycles of denaturation at 95 °C for 5s and annealing and extension at 58 °C for 20s. A standard curve was obtained by using COG2 amplicon-containing plasmid in serially diluted tenfold from 10^7^ to 1 genomic copy. Amplification was performed on CFX96 Real-Time System (Bio-Rad), and data were collected and analyzed with Bio-Rad CFX Manager 2.0.

### Assessment of HuNoV diarrhea

Daily rectal swabs were collected following HuNoV inoculation, fecal consistency was scored based on our previous system^22^. From PID1 to PID17, pigs were scored as follows: 0, solid; 1, semisolid; 2, pasty; 3, semiliquid; and 4, liquid. From PID18 to PID28, pigs were scored based on a more stringent system since the feces from older pigs were looser: 0, solid; 1, pasty; 2, semiliquid; and 3, liquid. Pigs with daily fecal scores of 2 or greater were considered diarrheic.

### Immunohistostaining

Sections of intestinal tissues were collected during necropsies, fixed overnight in 10% neutral formalin, embedded in paraffin, and sectioned 5 μm on positively charged glass slides. For immunostaining, slides were deparaffinized and rehydrated via graded ethanol series. Enzymatic antigen retrieval was performed by digesting sections in proteinase K solution (80 μg ml^−1^, Sigma Aldrich) at 37 °C for 30 min. Slides were incubated in blocking buffer (tris-buffered saline (TBS) with 10% normal pig serum and 1% bovine serum albumin (BSA)) at room temperature for 2 h. For immunostaining of duodenum and jejunum, the staining procedure consisted of incubation with a goat anti-HuNoV GII.4 VLP polyclonal antibody diluted in TBS containing 1% BSA overnight at 4 °C, washing with TBS containing 0.025% Triton X-100, and incubation with Alexa Fluor 488-labeled donkey anti-goat secondary antibody (A-11055; Thermo Fisher Scientific; 1:500) diluted in TBS containing 1% BSA at room temperature for 1 h. For immunostaining of mesentery, mouse anti-CD79 (VP-C366; Vector Laboratories; 1:1000) and Alexa Fluor 546-labeled donkey anti-mouse secondary antibody (A10036; Thermo Fisher Scientific; 1:500) were used in the two incubation steps above. Slides were washed in TBS and mounted in Vectashield containing DAPI to stain cell nuclei (Vector Laboratories). Images were acquired on a Zeiss LSM 880 confocal laser scanning microscope with Zen software (Zeiss).

### Statistics

All pigs infected with HuNoV were randomly assigned to be euthanized on PID3, PID10, or PID28. For virus shedding, diarrhea scores, and virus in blood, pigs euthanized on PID28 contributed data to PID3 and PID10 subgroups, as did PID10 contribute data to PID3 subgroup. Statistical analysis (Mann-Whitney test or Student’s *t*-test as specified) was performed using GraphPad Prism 6.0 (GraphPad Software). *P* value < 0.05 was considered statistically significant.

## Additional Information

**How to cite this article**: Lei, S. *et al.* Increased and prolonged human norovirus infection in RAG2/IL2RG deficient gnotobiotic pigs with severe combined immunodeficiency. *Sci. Rep.* **6**, 25222; doi: 10.1038/srep25222 (2016).

## Supplementary Material

Supplementary Information

## Figures and Tables

**Table 1:** Incidence of clinical signs and fecal virus shedding in Gn pigs.

Group	Time	*n*	Diarrhea		Virus shedding	
			Pigs with diarrhea (%)*	Mean duration days (SEM)**	Pigs shedding virus (%)*	Mean duration days (SEM)**
WT	PID1–3	11	8 (73%)	1.0 (0.3)	9 (82%)	1.5 (0.3)
RAG2/IL2RG deficiency		12	6 (50%)	0.8 (0.3)	11 (92%)	1.3 (0.2)
WT	PID4–10	8	7 (88%)	2.8 (0.6)	7 (88%)	3.0 (0.7)
RAG2/IL2RG deficiency		9	6 (67%)	1.7 (0.6)	9 (100%)	3.4 (0.3)
WT	PID11–17	5	5 (100%)	2.6 (1.0)	2 (40%)	1.2 (1.0)^A^
RAG2/IL2RG deficiency		4	4 (100%)	4.5 (1.2)	4 (100%)	5.5 (0.6)^B^
WT	PID18–28	5	1 (20%)	0.4 (0.4)	0^A^	0^A^
RAG2/IL2RG deficiency		4	2 (50%)	0.8 (0.5)	4 (100%)^B^	4.5 (0.3)^B^

Gn pigs were inoculated with a HuNoV GII.4 2006b variant 092895 at 6–7 days of age. Rectal swabs were collected daily after inoculation to determine diarrhea and virus shedding. SEM, standard error of the mean. *Fisher’s exact test or **Mann-Whitney test was used for statistical analysis. Groups with significant differences (*P* < 0.05) were indicated with letters A and B.
