# Supplementary material for: Increased and prolonged human norovirus infection in RAG2/IL2RG deficient gnotobiotic pigs with severe combined immunodeficiency
Source: Sci Rep. 2016 Apr 27;6:25222. doi: 10.1038/srep25222 (PMC4846862; doi:10.1038/srep25222)
Supplement: Supplementary Information [file srep25222-s1.pdf]

Supplementary Information for

Increased and prolonged human norovirus infection in RAG2/IL2RG deficient gnotobiotic pigs with severe combined immunodeficiency

Shaohua Lei[1,*], Junghyun Ryu[2,*], Ke Wen[1], Erica Twitchell[1], Tammy Bui[1], Ashwin Ramesh[1], Mariah Weiss[1], Guohua Li[1], Helen Samuel[1], Sherrie Clark-Deener[3], Xi Jiang[4], Kiho Lee[2,+], Lijuan Yuan[1,+]

[1]Department of Biomedical Sciences and Pathobiology, Virginia-Maryland College of Veterinary Medicine, Virginia Tech, Blacksburg, VA 24061, USA.

[2]Department of Animal and Poultry Sciences, College of Agriculture and Life Sciences, Virginia Tech, Blacksburg, VA 24061, USA.

[3]Department of Large Animal Clinical Sciences, Virginia-Maryland College of Veterinary Medicine, Virginia Tech, Blacksburg, VA 24061, USA.

[4]Division of Infectious Diseases, Cincinnati Children's Hospital Medical Center, Cincinnati, OH 45229, USA.

[*]These authors contributed equally to this work.

[+]Correspondence and requests for materials should be addressed to K.L. (email: kiholee@vt.edu) or L.Y. (email: lyuan@vt.edu).

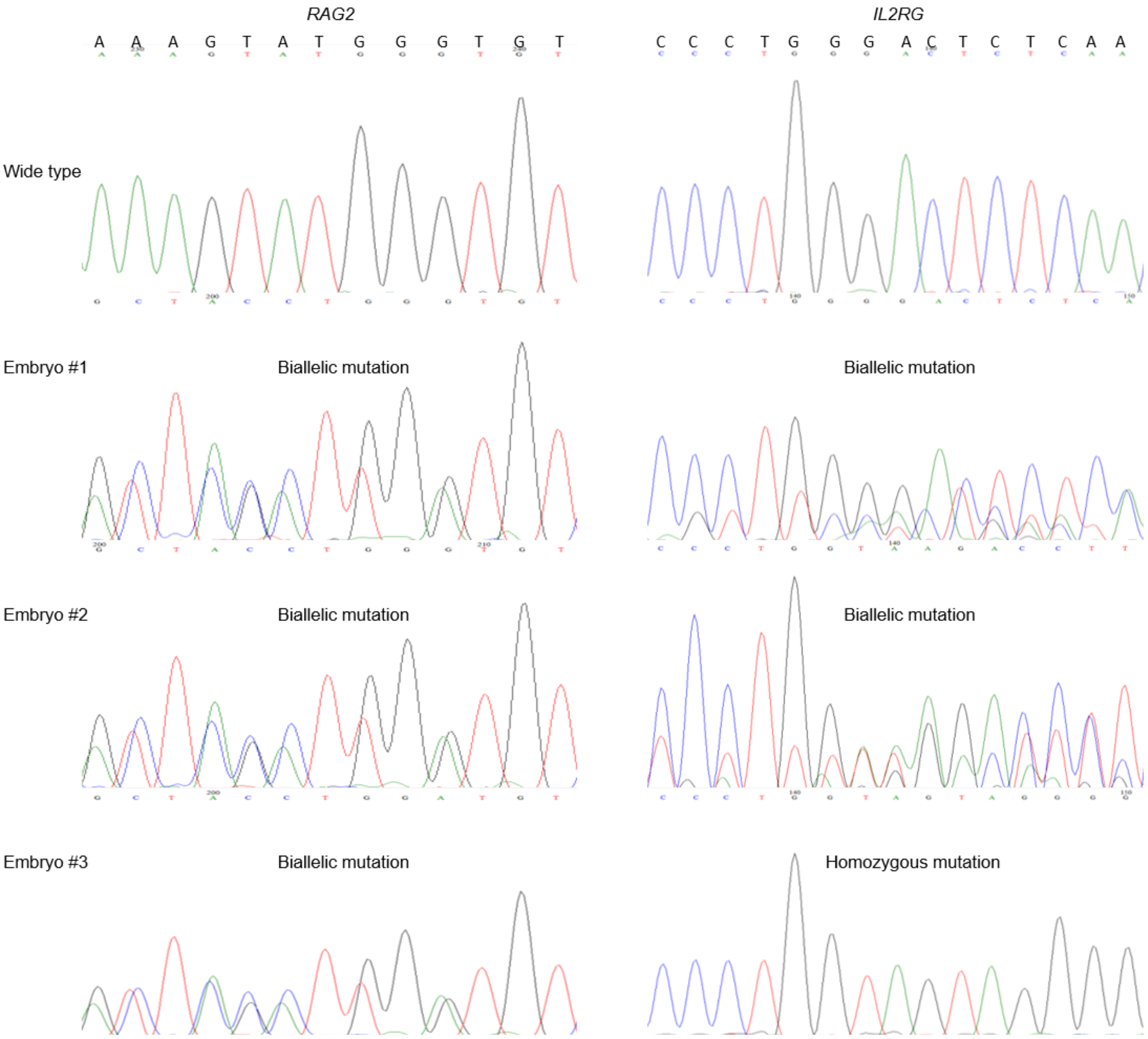

Supplementary Figure 1. Types of genetic mutations induced by CRISPR/Cas9 system. Representative images of sequencing readings from individual blastocysts showing mutations on *RAG2* and *IL2RG*.

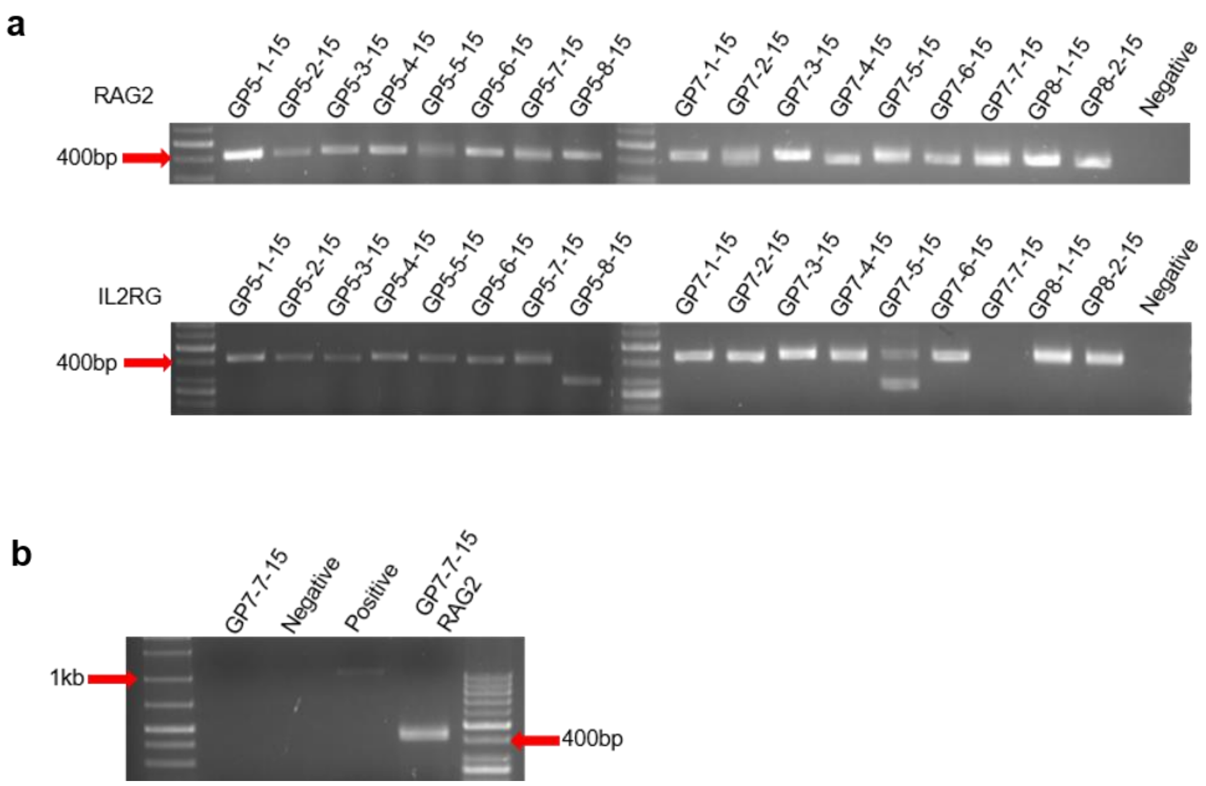

Supplementary Figure 2. PCR amplicons to genotype RAG2/IL2RG deficient pigs. (**a**) Gel images showing PCR products which flank projected cutting sites of *RAG2* and *IL2RG* by the CRISPR/Cas9 system; *IL2RG* was not amplified from genomic DNA of pig GP7-7-15. Genomic DNA templates of newborn pigs were purified from tail tissues, which were obtained at 1 day of age. (**b**) PCR amplification to identify larger deletion of IL2RG in pig GP7-7-15. There was no amplification of *IL2RG* from pig GP7-7-15 using a primer set that amplifies 417 bp from WT pig DNA. When a new set of primers were used to amplify over 1kb of IL2RG, still no PCR product was detected. A fragment of *RAG2* was amplified from the same genomic DNA, indicating the quality of the genomic DNA was not compromised. A wild type pig genomic DNA was used as positive control PCR.

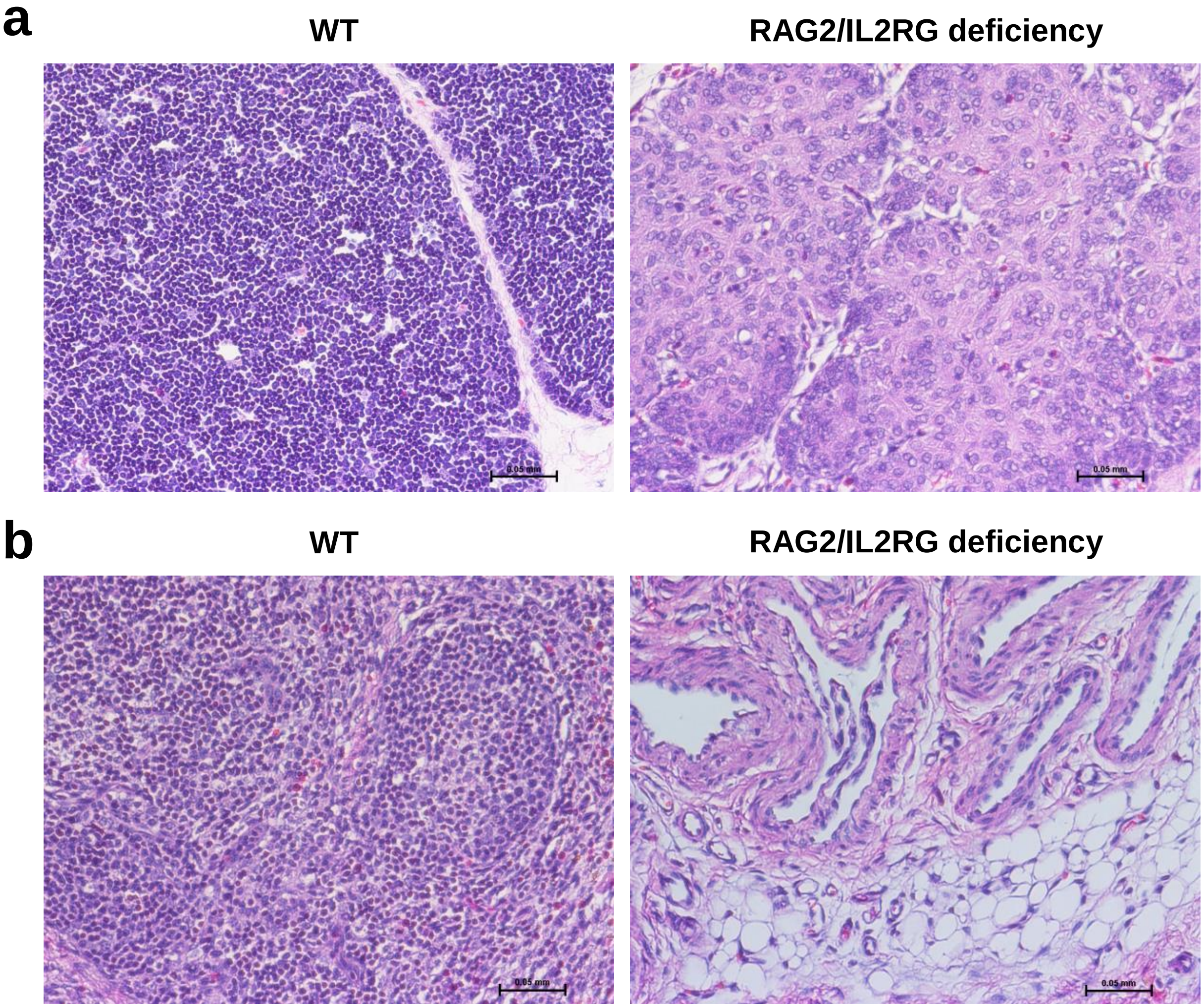

Supplementary Figure 3. Lack of lymphocytes in thymus and mesentery in some RAG2/IL2RG deficient pigs. (**a**) Representative images of H&E stained sections showing lack of lymphocytes within observed thymus in a RAG2/IL2RG deficient pig (right), while thymuses were populated with lymphocytes in WT pigs (left). (**b**) Representative images of H&E stained sections showing lack of lymphocytes within mesenteric tissue in a RAG2/IL2RG deficient pig (right), while mesenteric lymph nodes were populated with lymphocytes in WT pigs (left). Scale bar, 50 µm.

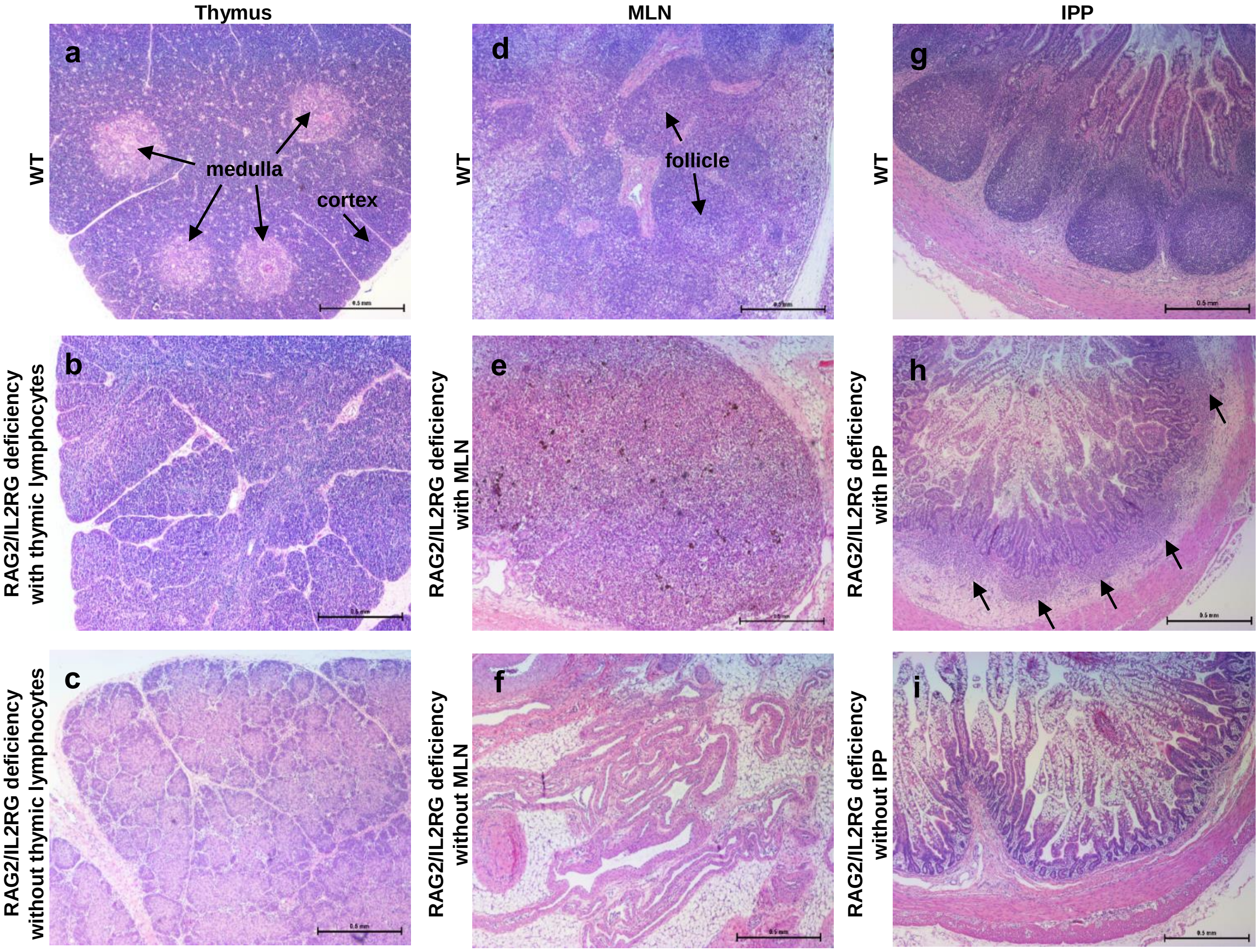

Supplementary Figure 4. Abnormal morphology of thymus, MLN, and IPP in RAG2/IL2RG deficient pigs. Representative images of H&E stained sections showing abnormal structure of thymus (left panel), MLN (middle panel), and IPP (right panel) in RAG2/IL2RG deficient pigs at 34 days of age. (**a**) Histologically normal thymus from a WT pig with defined cortex and medulla. Thymus from RAG2/IL2RG deficient pigs with lymphocytes but no distinction between cortex and medulla (**b**) or without lymphocytes (**c**). (**d**) Histologically normal MLN from a WT pig. **(e)** MLN from RAG2/IL2RG deficient pigs without defined cortical or medullary lymphoid tissue. (**f**) Mesentery without MLN from a RAG2/IL2RG deficient pig. (**g**) Normal IPP from WT pigs. (**h**) Poorly developed and unstructured small IPP indicated by arrows in RAG2/IL2RG deficient pigs. (**i**) Ileum without IPP from a RAG2/IL2RG deficient pig. Scale bar, 0.5 mm.

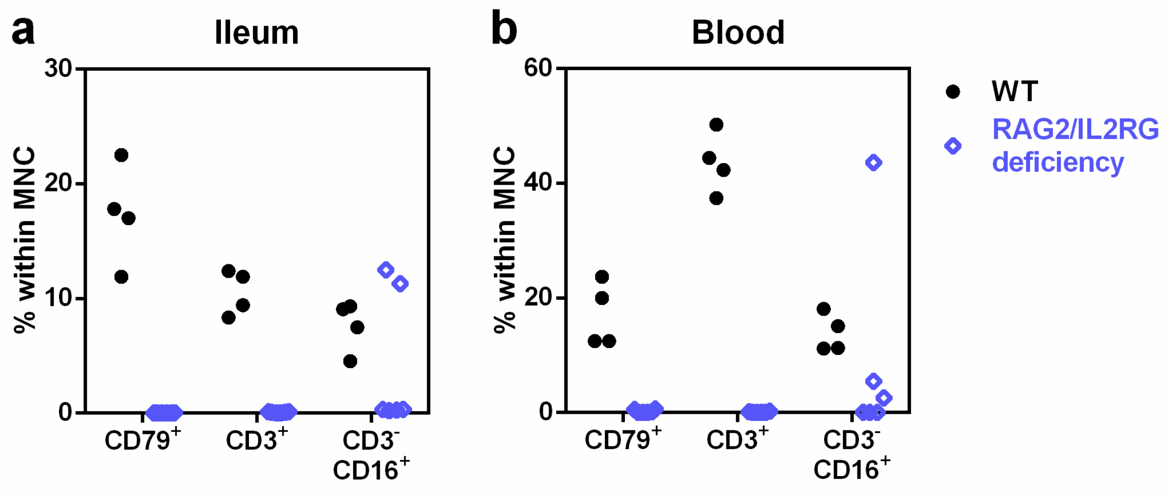

Supplementary Figure 5. Proportion of B cells (CD79$^+$), T cells (CD3$^+$), and NK cells (CD3$^-$ CD16$^+$) within MNC. The frequency of B cells, T cells, and NK cells within MNC from ileum (**a**) and blood (**b**) were quantified by flow cytometry for WT (n = 4) and RAG2/IL2RG deficient pigs (n = 6). Data are presented as individual animal data points.

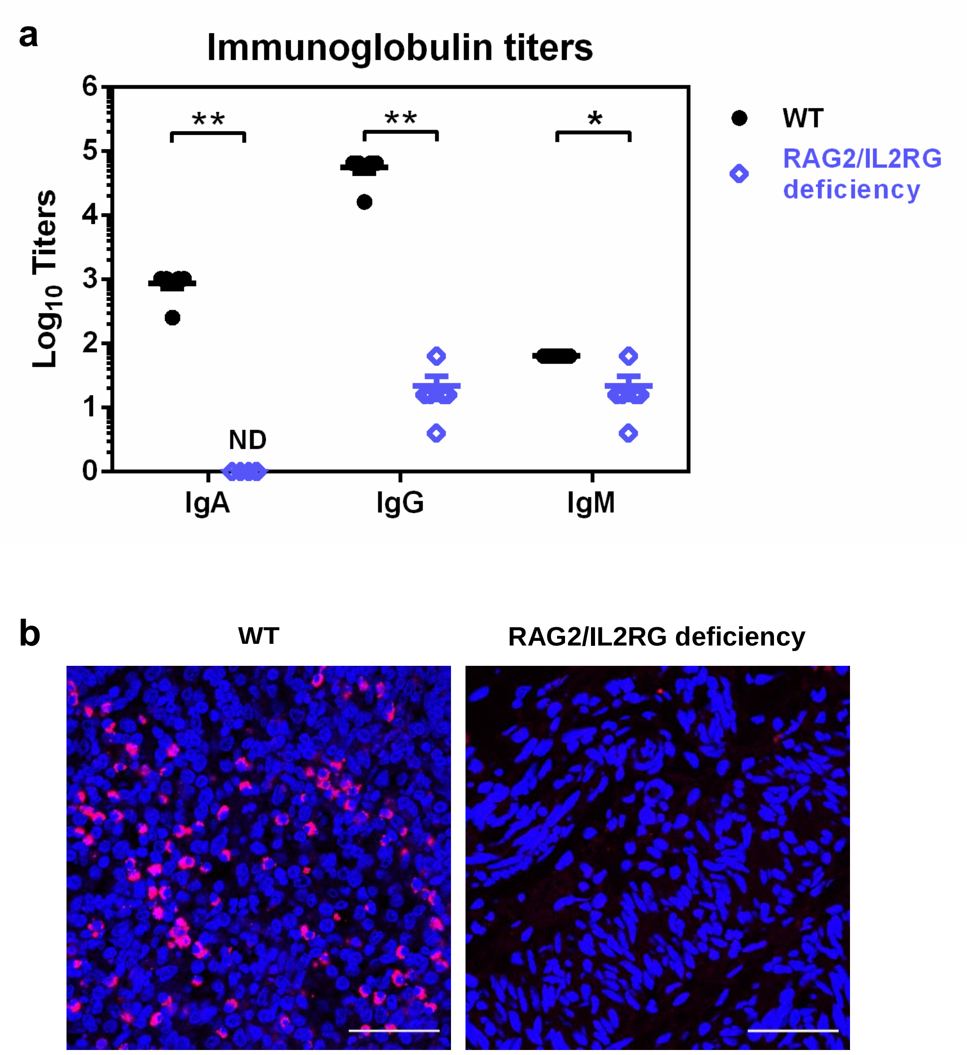

Supplementary Figure 6. Depletion of B cells in RAG2/IL2RG deficient pigs at 34 days of age. (**a**) Immunoglobulin titers in serum were determined by ELISA in WT (n = 5) and RAG2/IL2RG deficient pigs (n = 6). Data are presented as means ± s.e.m. with individual animal data points. Statistical significance was determined by Mann-Whitney test. *P<0.05, **P<0.01. **(b)** Mesentery tissue sections from WT and RAG2/IL2RG deficient pigs were stained to detect B cells ($CD79^+$, red) with counter staining of cell nuclei (DAPI, blue). Scale bar, 50 μm.

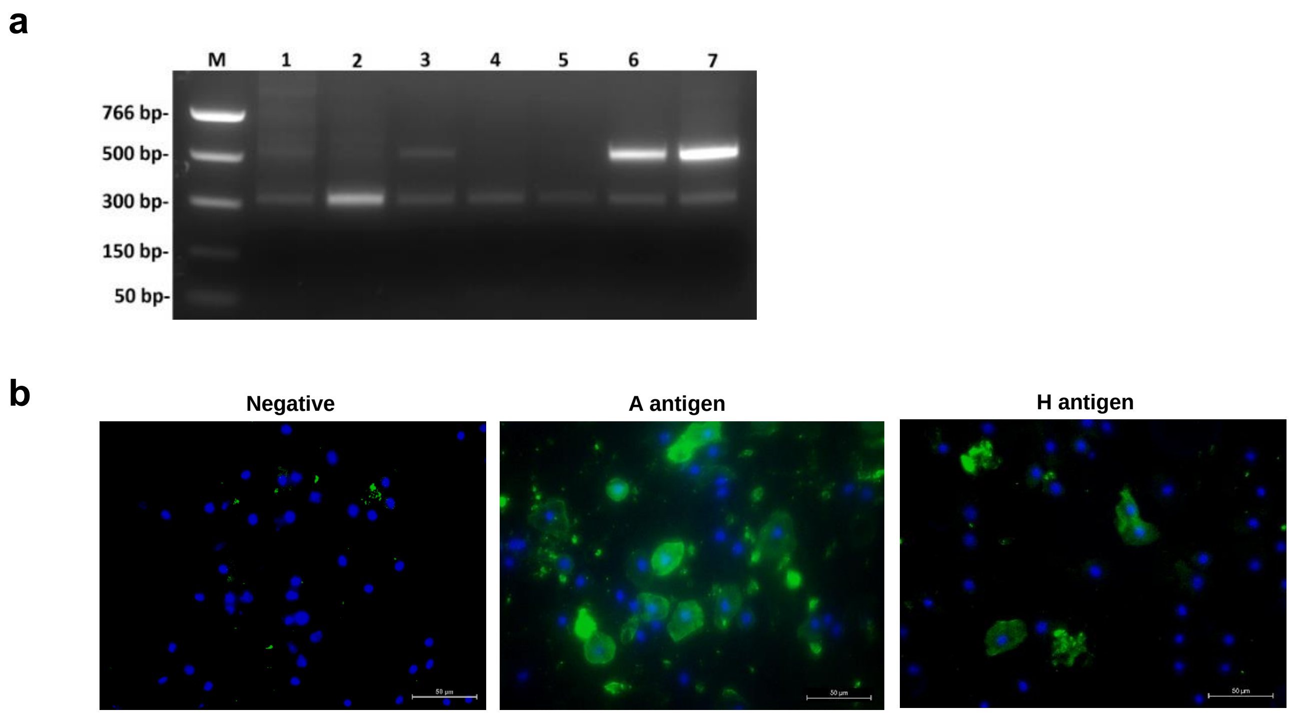

Supplementary Figure 7. HBGA typing of Gn pigs by PCR and immunofluorescence assay. (**a**) Pigs were determined to be A^+ or A^- by PCR using genomic DNA from blood. Representative gel image showing A^+ samples with a 500 bp PCR product in addition to a 300 bp PCR internal control (Lane 1, 3, 6, 7). (**b**) Buccal cells were fixed on glass slides and stained for A or H antigen (FITC, green) and nuclei (DAPI, blue). Representative images indicate HBGA A^- and H^-, A^+, and H^+ pigs. Scale bar, 50 µm.

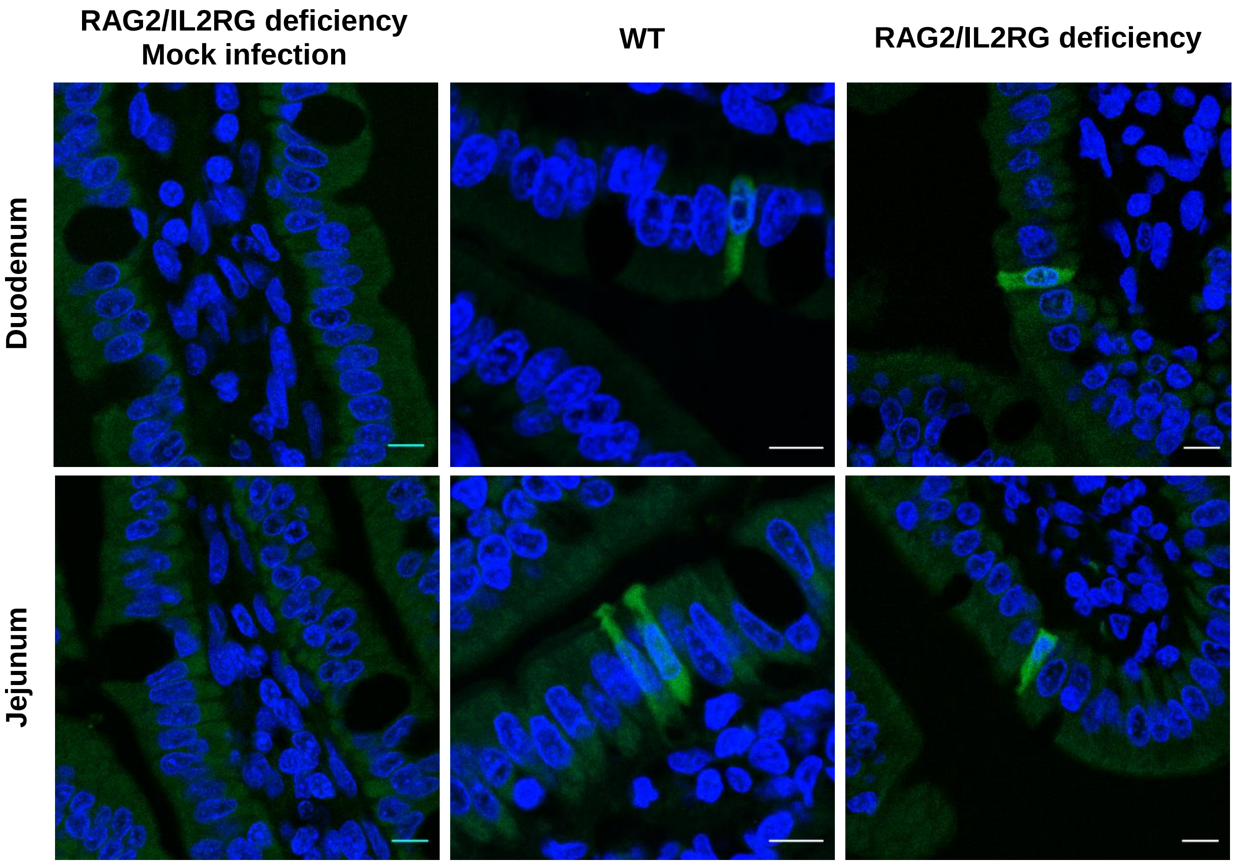

Supplementary Figure 8. HuNoV infection of enterocytes in Gn pigs. Immunohistochemistry of duodenum (upper panel) and jejunum (lower panel) from pigs euthanized on PID3 for HuNoV capsid protein (bright green) and cell nuclei (blue). Representative images showing HuNoV infection of enterocytes in WT and RAG2/IL2RG deficient pigs. Scale bar, 10 µm.

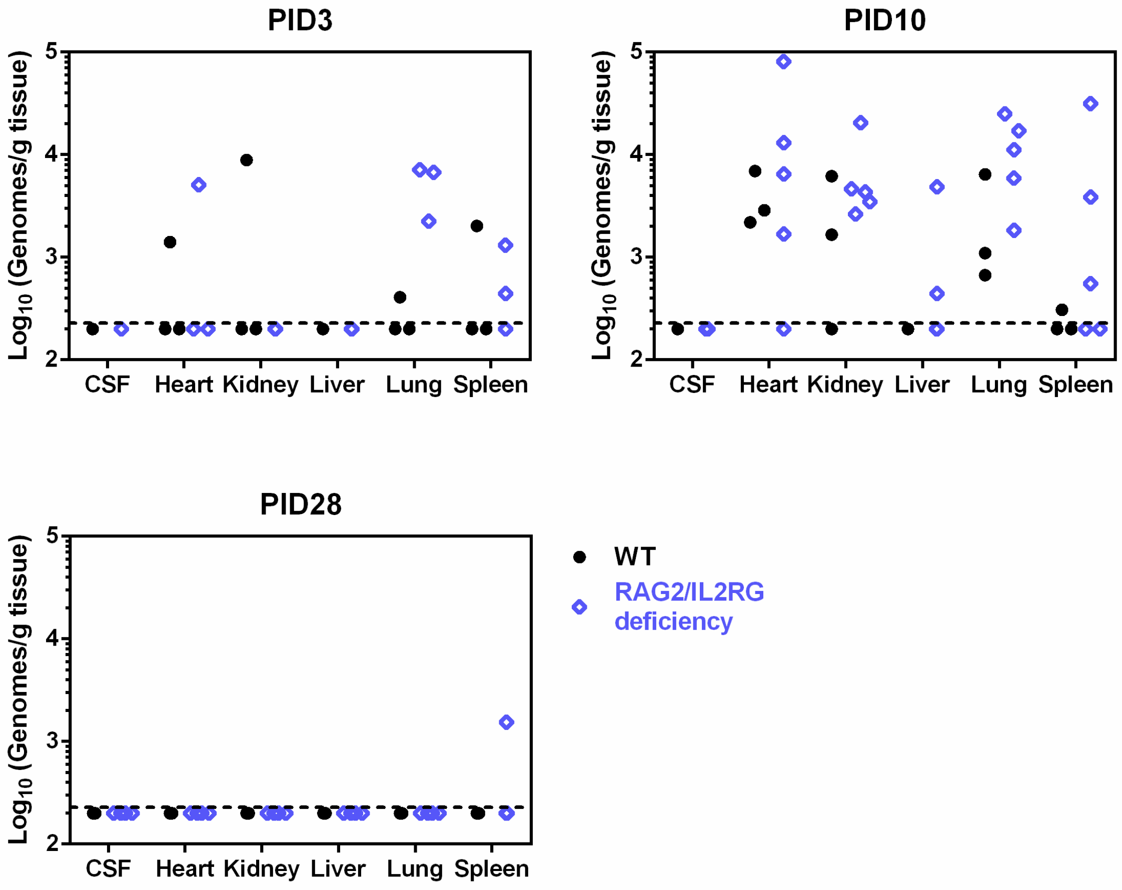

Supplementary Figure 9. HuNoV genomes in extraintestinal tissues in Gn pigs. HuNoV genomes in CSF (cerebrospinal fluid), heart, kidney, liver, lung, and spleen in WT and RAG2/IL2RG deficient pigs euthanized on PID3, PID10, and PID28 were measured by qRT-PCR. WT groups, PID3 *n*=3, PID10 *n*=3, PID *n*=5; RAG2/IL2RG deficiency groups, PID3 *n*=3, PID10 *n*=5, PID *n*=4. Dashed line indicates limit of detection. Data are presented as individual animal data points.

Supplementary Table 1. Optimization of CRISPR/Cas9 system to induce mutations during embryogenesis. To minimize potential cytotoxicity, CRISPR/Cas9 RNAs were introduced into pig zygotes at four different concentrations, and their effectiveness of introducing mutations on *RAG2* was examined by genotyping blastocysts. Concentrations of 2.5 ng μl^{-1} sgRNA and 5 ng μl^{-1} Cas9 mRNA was used for all subsequent studies.

Concentration of CRISPR/Cas9 (ng/µl)	Total # of embryos injected	% of blastocyst on day 7 (number of blastocysts/cleaved)	# of blastocyst genotyped	Genotypes				
				Homozygous mutation	Biallelic mutation	Mosaic mutation	Heterozygous mutation	WT
10/20	49	18.91% (7/37)	5	2	2	1	0	0
2.5/5	41	30.00% (9/30)	4	1	3	0	0	0
1/2	42	41.66% (10/24)	4	0	1	1	0	2
0.5/1	41	33.33% (10/30)	5	0	2	0	3	0
0/0 (water)	39	41.93% (13/31)	N/A	N/A	N/A	N/A	N/A	N/A

Supplementary Table 2. *In vitro* targeting of *RAG2* and *IL2RG*. Using the optimized concentration of sgRNA and Cas9 mRNA, *RAG2* and *IL2RG* were targeted simultaneously *in vitro*. Among the six genotyped blastocysts, mutations on both *RAG2* and *IL2RG* were detected, and no wild type sequence was found.

Targeted gene	Total # of embryos injected	Cleaved	% of blastocyst on day 7 (number of blastocyst/cleaved)	# of blastocyst genotyped	Genotypes				
					Homozygous mutation	Biallelic mutation	Mosaic mutation	Heterozygous mutation	WT
RAG2	200	155	36.7% (57/155)	6	0	5	1	0	0
IL2RG					2	4	0	0	0

Supplementary Table 3. Generation of RAG2/IL2RG deficient pigs. Five embryo transfers were conducted on day 5 or 6 using five surrogate sows, a total of seventeen piglets were generated from the three pregnant surrogates.

Surrogate ID	# of embryos generated	# of embryos transferred into a recipient	Pregnancy	# of piglets
342	318	70 (day 6)	No	-
343	308	132 (day 5)	Yes	8
347	301	77 (day 6)	No	-
356	283	151 (day 5)	Yes	7
348	295	165 (day 5)	Yes	2

Supplementary Table 4. Genotyping result of RAG2/IL2RG deficient pigs. Bold letters indicate insertion or change in nucleotides, and '-' indicates deletion of a nucleotide. All piglets carried mutations on both *RAG2* and *IL2RG*.

**Pig ID**	***RAG2***		***IL2RG***	
	DNA	Predicted Amino Acid	DNA	Predicted Amino Acid
Wild type	TGATGTCGTGTATAGTCGAGGGAAAAGTATGGGTGTTCTCTTT	+/+	CTCTCTACACCCCCTGGGACTCTCAACGTTTCCACTCTACCCC	+/+ or +/ Y
GP5-1-15	TGATGTCGTGTATAGTCGAGGG-----------------------TGTTCTCTTT<br>TGATGTCGTGTATAGTCGAGGG---------------------------TTCTCTTT	Δ150-527/ Δ150-527	CTCTCTACACCCCCT--GGACTCTCAACGTTTCCACTCTACCCC	Δ49-368/ Δ49-368
GP5-2-15	TGATGTCGTGTATAGTC-------------------GTATGGGTGTTCTCTTT	Δ149-151/ Δ149-151	CTCTCTACACC—**AC**----------CTCTCAACGTTTCCACTCTACCCC	Δ48, 49/ Y
GP5-3-15	TGATGTCGTGTATAGTCGAGGG**A**AAAAGTATGGGTGTTCTCTTT	Δ151-527/ Δ151-527	CTCTCTACACCCC-------------CTCTCAACGTTTCCACTCTACCCC	Δ48, 49/ Y
GP5-4-15	TGATGTCGTGTATAGTCGAGGG**A**AAAAGTATGGGTGTTCTCTTT	Δ151-527/ Δ151-527	CTCTCTACACCCCCTG**T**GGACTCTCAACGTTTCCACTCTACCCC<br>CTCTCTACACCCCCTGG**AA**ACTCTCAACGTTTCCACTCTACCCC	Δ47-368 / Δ48-368
GP5-5-15	TGATGTCGTGTAT------------------------------------GGGTGTTCTCTTT<br>TGATGTCGT------------------------------------GTATGGGTGTTCTCTTT<br>TGATGTCGTGTATAGTCGAGGG**A**AAAAGTATGGGTGTTCTCTTT	Δ147-527/ Δ147-527/ Δ151-527	CTCTCTACACCCC—**T**--**AC**ACTCTCAACGTTTCCACTCTACCCC	Δ47-368/ Δ47-368
GP5-6-15	TGATGTCGTGTATAGTCGAGGG--AAAGTATGGGTGTTCTCTTT<br>TGATGTCGTGTATAGTCGAGGGAAAA--TATGGGTGTTCTCTTT	Δ151-527/Δ151-527	CTCTCTACACC-----**T**----------CTCTCAACGTTTCCACTCTACCCC	Δ47-368/ Y
GP5-7-15	TGATGTCGTGTATAGTC---TCTCTTT<br>TGATGTCGTGTATAGTCGA------------------------GGGTGTTCTCTTT	Δ148-527/Δ151-527	CTCTCTACACCCC-------------------TCAACGTTTCCACTCTACCCC<br>CTCTCTACACCCCCT--GGACTCTCAACGTTTCCACTCTACCCC	Δ47-49/ Δ49-368/ Y
GP5-8-15	TGATGTCGTGTATAGTCGAGGG**A**AAAAGTATGGGTGTTCTCTTT	Δ151-527/ Δ151-527	**27bp deletion** -GGG**A**ACTCTCAACGTTTCCACTCTACCCC	Δ39-368/ Δ39-368
GP7-1-15	TGATGTCGTGTATAGTCGAGGG**AAAA**AAAAGTATGGGTGTTCTCTTT	Δ151-527/ Δ151-527	CTCTCTACACCCCCT--GGACTCTCAACGTTTCCACTCTACCCC	Δ49-368/ Y
GP7-2-15	TGATGTCGTGTATAGTCGAGGGA**GAACACCCATA**TGTTCTCTTT<br>TGATGTCGTGTATAGTCGA------------------------**28bp deletion**--	Δ150-527/ Δ149-527	CTCTCTACACCCCCT-----------------CAACGTTTCCACTCTACCCC<br>CTCTCTACACCCC---------------TCTCAACGTTTCCACTCTACCCC	Δ48-368/ Δ47-368/ Y
GP7-3-15	TGATGTCGTGTATAGTCGAGGGA**CT**AAAGTATGGGTGTTCTCTTT<br>TGATGTCGTGTATAGTCGAGGGAAA**TAT**AGTATGGGTGTTCTCTTT	Δ150-527/ 151Y	CTCTCTACAC**TCCTGCTCTCTACACC**CTCTCAACGTTTCCACTCTACCCC<br>CTCTCTACACCCCCTGG--ACTCTCAACGTTTCCACTCTACCCC	Δ41-368/ Δ49-368
GP7-4-15	TGATGTCGT------------------------------------GTATGGGTGTTCTCTTT	Δ147-527/ Δ147-527	CTCTCTACACCCCCTGGG**GTGTAC**ACTCTCAACGTTTCCACTCTACCCC<br>CTCTCTACACCCCCTGGG**GTGTACC**CTCTCAACGTTTCCACTCTACCCC	49Y, 50Y / T49Y, 50Y, 51P
GP7-5-15	TGATGTCGTGTATAGTCGAGG-------------TATGGGTGTTCTCTTT<br>TGATGTCGTGTATAGTCGAGGG**TGTTCCCAACCCA**GGGTGTTCTCTTT	Δ150, 151/ Δ150-527	CTCTCTACACCCCCTG**TTGA**GACTCTCAACGTTTCCACTCTACCCC<br>-----------------------------45bp deletion---------------------------ACTCTACCCC	48V, G49E/ Δ39-53
GP7-6-15	TGATGTCGTGTATAGTCG----------------A**C**TATGGGTGTTCTCTTT	Δ149-527/ Δ149-527	CTCTCTACACCCC------------CTCTCAACGTTTCCACTCTACCCC	Δ48, 49/ Δ48, 49
GP7-7-15	TGATGTCGTGTATAGTCGA----------------------GGGTGTTCTCTTT<br>TGATGTCGTGTATAGTCGAGG------------TATGGGTGTTCTCTTT	Δ150-527/ Δ150, 151	No amplification	N/A
GP8-1-15	TGATGTCGTGTATAGTCGAGGGAAA**G**AGTATGGGTGTTCTCTTT<br>TGATGTCGTGTATAGTCGAGGGAAAA**AA**GTATGGGTGTTCTCTTT	Δ151-527/ Δ151-527	CTCTCTACACCCCCTGG**TA**GACTCTCAACGTTTCCACTCTACCCC<br>CTCTCTAC----------------------ACTCTCAACGTTTCCACTCTACCCC	Δ49-368/ Δ46-368
GP8-2-15	TGATGTCGTGTATAGTCGAGGG**A**AAAAGTATGGGTGTTCTCTTT	Δ151-527/ Δ151-527	CTCTCTACAC----------------------TCTCAACGTTTCCACTCTACCCC<br>CTCTCTACACCCC-------------CTCTCAACGTTTCCACTCTACCCC	Δ46-368/ Δ48, 49

Supplementary Table 5. Genotypes and phenotypes of RAG2/IL2RG deficient pigs.

Pig ID	Gender	*RAG2*	*IL2RG*	Thoracic thymus[a]	Cervical thymus[a]	MLN[b]	IPP[c]	HBGA type[d]	General health	Age on euthanasia
Gp5-2-15*	M	Homozygous[#]	Hemizygous[#]	+	-	++	+	H	Normal	9 days (PID3)
Gp5-3-15*	M	Homozygous	Hemizygous[#]	-	-	++	+	H	Normal	9 days (PID3)
Gp5-4-15*	F	Homozygous	Biallelic	-	+, ND[e]	-	-	A	Normal	9 days (PID3)
Gp5-5-15*	F	Mosaic	Homozygous	-	-	-	-	H	Normal	16 days (PID10)
Gp5-7-15*	M	Biallelic	Mosaic[#]	++	-	++	+	H	Normal	16 days (PID10)
Gp5-8-15*	F	Homozygous	Homozygous	-	-	-	-	A	Normal	9 days (mock)
Gp7-1-15*	M	Homozygous	Hemizygous	+, ND	++, ND	-	-	A	Normal	34 days (PID28)
Gp7-2-15*	M	Biallelic	Mosaic	++, ND	++, ND	-	-	H	Normal	34 days (PID28)
Gp7-3-15*	F	Biallelic[#]	Biallelic	+, ND	+, ND	-	-	A	Normal	34 days (PID28)
Gp7-4-15*	F	Homozygous	Biallelic[#]	++	++	+	+	H	Normal	34 days (PID28)
Gp7-5-15*	F	Biallelic[#]	Biallelic[#]	++	+	+	+	A	Normal	17 days (PID10)
Gp7-6-15*	F	Homozygous	Homozygous[#]	++	+	+	+	H	Normal	17 days (PID10)
Gp7-7-15*	M	Biallelic[#]	Hemizygous	-	-	-	-	H	Normal	17 days (PID10)
Gp5-1-15	F	Biallelic	Homozygous	-	+, ND	-	-		Normal	9 days (PID3)
GP5-6-15	M	Biallelic	Hemizygous	-	-	-	-		Failure to thrive	3 days
Gp8-1-15	F	Biallelic	Biallelic	-	+, ND	-	-		Normal	34 days (PID28)
Gp8-2-15	F	Homozygous	Biallelic[#]	++	+	++	-		Normal	34 days (PID28)

* Pigs used in this HuNoV infection study. [#] Pre-mature stop codon was not generated on at least one allele based on genotyping.

[a] -, not observed; +, smaller than wild type pigs (≤ 15 mm); ++, similar to wild type pigs (15 mm to 25 mm).

[b] MLN, mesenteric lymph nodes; -, not observed; +, less than wild type pigs; ++, similar to wild type pigs.

[c] IPP, ileal Peyer's patches; -, not observed; +, poorly developed and unstructured small IPP.

[d] Pigs used in this study were blood-typed as A^+ or H^+ by PCR and/or immunofluorescence assay.

[e] ND, lymphocytes were not detected in thymus as indicated by H&E staining, only epithelial components were observed.

Supplementary Table 6. Primers and HuNoV qRT-PCR probe used in this study.

Name	Sequence (5'- 3')	Product size
**To introduce sgRNA into px330**		
RAG2 F1	CAC CGT ATA GTC GAG GGA AAA GTA	
RAG2 R1	AAA CTA CTT TTC CCT CGA CTA TAC	
RAG2 F2	CAC CGA AGG CAG ATA TGG TCA TTC	
RAG2 R2	AAA CGA ATG ACC ATA TCT GCC TTC	
IL2RG F1	CAC CGG AAA CGG TTG AGA GTC CCA	
IL2RG R1	AAA CCT GGG ACT CTC AAC GTT TCC	
IL2RG F2	CAC CGT GGA AAC GTT GAG AGT CCC	
IL2RG R2	AAA CGG GAC TCT CAA CGT TTC CAC	
**To generate template DNA for *in vitro* transcription of sgRNA and mRNA form of Cas9**		
T7 RAG2 F1	TTA ATA CGA CTC ACT ATA GGT ATA GTC GAG GGA AAA GTA	
T7 RAG2 F2	TTA ATA CGA CTC ACT ATA GGA AGG CAG ATA TGG TCA TTC	
T7 IL2RG F1	TTA ATA CGA CTC ACT ATA GGG AAA CGG TTG AGA GTC CCA	
T7 IL2RG F2	TTA ATA CGA CTC ACT ATA GGT GGA AAC GTT GAG AGT CCC	
T7 sgRNA R1	AAA AGC ACC GAC TCG GTG CC	
Cas9 F	TAA TAC GAC TCA CTA TAG GGA GAA TGG ACT ATA AGG ACC ACG AC	
Cas9 R	GCG AGC TCT AGG AAT TCT TAC	
**To genotype *RAG2* and *IL2RG* mutations introduced by CRISPR/Cas9 system**		
RAG2 F	AAG GAT TCC TGC TAC CTT CCT CCT	426bp
RAG2 R	AGA TAG CCC ATC TTG AAG TTC TGG	
IL2RG F	CTG GAC TAT TAG AAG GAT GTG GGC	417bp
IL2RG R	ATA TAG TGG GAA GCC TGG GAT GCT	
IL2RG extend F	GAT TAA CAC CTA ATC TCC CAG AGG ATT TAG CCT GTG TC	1017bp
IL2RG extend R	CCT CTT TTC CAA ACC AAC AGC CAG AAG TGA TC	
**HBGA typing of pigs**		
ABO4s	AGCTGTTCCTGGAGACAGCGGAGA	500bp
ABO5a	CAGGTGGCTCTCATCATGCCACAC	
Pig5	CCCTGGAACTCTGCCACTGTC	300bp
Pig3	CTGCACGTAGCACCAGGGTCT	
**To detect HuNoV genomes**		
COG2F	CARGARBCNATGTTYAGRTGGATGAG	
COG2R	TCGACGCCATCTTCATTCACA	
probe RING2	/56-FAM/TGGGAGGGCGATCGCAATCT/3BHQ1/	
